# Can Generative AI and ChatGPT Break Human Supremacy in Mathematics and Reshape Competence in Cognitive-Demanding Problem-Solving Tasks?

**DOI:** 10.3390/jintelligence13040043

**Published:** 2025-04-02

**Authors:** Deniz Kaya, Selim Yavuz

**Affiliations:** 1 Department of Mathematics Education, Faculty of Education, Nevsehir Hacı Bektas Veli University, 50300 Nevsehir, Türkiye; 2 Department of Curriculum and Instruction, School of Education, Indiana University Bloomington, Bloomington, IN 47405, USA; syavuz@iu.edu

**Keywords:** ChatGPT, cognitive load, generative artificial intelligence, NAEP, problem-solving, mathematics assessment, AI-driven education

## Abstract

This study investigates the potential of generative artificial intelligence tools in addressing cognitive challenges encountered by humans during problem-solving. The performance of ChatGPT-4o and GPT-4 models in the NAEP mathematics assessments was evaluated, particularly in relation to the cognitive demands placed on students. Sixty NAEP mathematics assessment tasks, coded by field experts, were analyzed within a framework of cognitive complexity. ChatGPT-4o and GPT-4 provided responses to each question, which were then evaluated using NAEP’s scoring criteria. The study’s dataset was analyzed using the average performance scores of students who answered correctly and the item-wise response percentages. The results indicated that ChatGPT-4o and GPT-4 outperformed most students on individual items in the NAEP mathematics assessment. Furthermore, as the cognitive demand increased, higher performance scores were required to answer questions correctly. This trend was observed across the 4th, 8th, and 12th grades, though ChatGPT-4o and GPT-4 did not demonstrate statistically significant sensitivity to increased cognitive demands at the 12th-grade level.

## 1. Introduction

The integration of generative artificial intelligence (GAI) technologies into educational environments has led to transformative changes in how students engage with learning materials and problem-solving processes. GAI offers advanced information processing capabilities, rapid feedback mechanisms, and adaptive learning strategies, making it a promising tool in situations where students experience cognitive load (Schorcht et al. 2024; Yoon et al. 2024; Zhai et al. 2024). By leveraging natural language processing and deep learning techniques, GAI can analyze student responses, guide learners through complex mathematical reasoning, and scaffold conceptual understanding. Prior research suggests that AI-driven tools can enhance higher-order thinking skills by structuring problem-solving tasks in ways that promote deeper cognitive engagement (Ayres 2006; Bommasani et al. 2021; Kalyuga 2011; Pesovski et al. 2024).

One of the major advantages of GAI in education is its ability to personalize learning experiences by tailoring instructional content to students’ strengths and weaknesses (Brown et al. 2020; Owoseni et al. 2024). Adaptive AI models dynamically adjust problem difficulty, provide targeted explanations, and generate step-by-step solutions, helping students advance at their own pace while receiving instant, data-driven feedback (Floridi and Chiriatti 2020). Additionally, instructors benefit from AI-driven analytics that assist in monitoring student progress, identifying learning gaps, and optimizing instructional strategies (Marcus and Davis 2019; Ogunleye et al. 2024).

Despite these advantages, the rapid rise of GAI in educational settings raises fundamental questions about the role of AI in cognitive problem-solving. If AI can match or even surpass human performance on tasks that require analytical reasoning, logical deduction, and conceptual understanding, then the goals of mathematics education—and the ways in which students develop these cognitive skills—may need to be re-evaluated (Zhai et al. 2024). A key concern is whether AI’s capabilities extend beyond computational efficiency to encompass genuine mathematical reasoning comparable to human cognition. Given this uncertainty, a critical need exists to examine how AI systems perform on standardized assessments that evaluate conceptual understanding rather than procedural computation.

This study aims to examine the capabilities of GAI in performing cognitively demanding mathematical tasks and assess its potential contributions to students’ problem-solving processes. While AI has demonstrated effectiveness in procedural problem-solving, its ability to handle tasks requiring conceptual reasoning, abstract thinking, and adaptive learning strategies remains uncertain. Addressing this gap is essential to understanding whether AI can function as a cognitive assistant that enhances students’ mathematical learning or if its limitations hinder its educational applicability. Additionally, this study seeks to provide guidance for integrating AI-driven technologies into educational assessments to ensure their responsible and effective use.

A central theoretical consideration in this research is the distinction between intelligence and rational problem-solving. As Stanovich (2014) emphasized, rationality and intelligence are distinct constructs, where rational problem-solving requires broader cognitive abilities beyond conventional intelligence metrics. This distinction is particularly relevant when evaluating GAI’s performance on tasks with varying cognitive demands, as it determines whether AI systems merely replicate pre-existing patterns or genuinely engage in problem-solving at a human level.

To analyze AI’s problem-solving capabilities, this study evaluates the performance of ChatGPT-4o and GPT-4 on the National Assessment of Educational Progress (NAEP) mathematics questions, which assess students’ understanding of mathematical concepts, logical reasoning, and applied problem-solving skills (Zhai and Pellegrino 2023). NAEP provides an effective benchmark for measuring AI’s mathematical competence across different grade levels, as it categorizes problems by cognitive complexity, allowing a systematic comparison between AI-generated solutions and student performance. Given these considerations, this study seeks to answer the following research questions:

RQ1. Are ChatGPT-4o and GPT-4 capable of scoring better than average human students of the same age group in NAEP mathematics tests?
RQ2. How do ChatGPT-4o and GPT-4 perform at different levels of cognitive demand in NAEP mathematics tests compared to average students of the same age group?

## 2. Literature Review

### 2.1. Cognitive Load Effects on Mathematics Problem-Solving Efficiency

In mathematics education, cognitive load theory (CLT) provides a framework for understanding how students allocate their limited cognitive resources while solving mathematical problems (Sweller 2011). CLT categorizes cognitive load into three distinct types: Intrinsic Cognitive Load (ICL), Extraneous Cognitive Load (ECL), and Germane Cognitive Load (GCL). ICL refers to the inherent difficulty of a mathematical problem, which depends on the complexity of the concepts involved and the learner’s prior knowledge (Sullivan et al. 2012). ECL is associated with how information is presented, and poorly structured problems or excessive extraneous information can overload students, making it harder for them to focus on the core mathematical principles (Paas et al. 2003). Finally, GCL represents the cognitive effort dedicated to meaningful learning and schema construction, which is necessary for long-term retention and mastery of complex problem-solving techniques (Sweller et al. 2011). A well-designed problem that encourages students to apply conceptual reasoning rather than memorization fosters a higher germane load, supporting deeper understanding (Paas and van Merriënboer 2020; Rittle-Johnson et al. 2009; van Gog et al. 2011; van Merriënboer and Sweller 2010).

AI-driven tutoring systems, such as ChatGPT, have the potential to manage cognitive loads effectively by minimizing extraneous demands and adapting instructional content based on the learner’s proficiency level. However, it remains uncertain whether AI can effectively differentiate between problem-solving processes that require deep conceptual understanding and those that rely on computational shortcuts. This study aims to explore how AI systems handle cognitive loads in problem-solving tasks and whether they can match human reasoning in tackling mathematically complex assessments.

### 2.2. GAI, AI, and ChatGPT Models

AI has undergone rapid advancements in machine learning and deep learning, leading to its widespread adoption in problem-solving and educational applications (Jordan and Mitchell 2015). AI systems can emulate human cognitive processes, analyze data patterns, and generate solutions across various domains (Zhai et al. 2020). Recent breakthroughs in GAI, such as ChatGPT-4o and GPT-4, have introduced more sophisticated natural language processing, reasoning, and problem-solving capabilities, making AI increasingly relevant in education (Bommasani et al. 2021).

Chatbots, particularly ChatGPT models, have been widely integrated into language processing, mathematics tutoring, and personalized learning platforms (Bommasani et al. 2021). GPT-4 outperforms its previous versions (GPT-1 [2018], GPT-2 [2019], GPT-3 [2020], and GPT-3.5 [2022]) in terms of language processing, logical reasoning, and problem-solving abilities (OpenAI 2023). The latest model, GPT-4o, incorporates multimodal processing capabilities, enabling it to analyze language, audio, images, and video, making it more adaptable to diverse educational needs (OpenAI 2024). Despite these improvements, questions remain about AI’s ability to handle complex, multi-step mathematical reasoning, which extends beyond computational fluency to conceptual problem-solving.

In mathematics education, ChatGPT has shown strong potential in breaking down complex problems into step-by-step solutions, providing instant feedback and personalized learning paths (Asare et al. 2023; Frieder et al. 2023; Sandu et al. 2024). Research suggests that ChatGPT can enhance student learning experiences by explaining abstract concepts, reducing cognitive load, and adapting to individual proficiency levels (Rahman and Watanobe 2023). However, its effectiveness declines as mathematical problems become more conceptually demanding. Studies indicate that, while ChatGPT excels at solving procedural and knowledge-based questions, its performance drops significantly on problems requiring deep reasoning and creative problem-solving (e.g., Dao and Le 2023; Frieder et al. 2023; Rahman and Watanobe 2023).

Empirical research has explored ChatGPT’s mathematical performance across different problem types. Wardat et al. (2023) predicted that ChatGPT’s capacity to solve advanced mathematical problems will improve over time but emphasized the need for careful pedagogical integration. Dao and Le (2023) found that ChatGPT achieved an 83% success rate on procedural mathematics tasks but struggled with problems requiring conceptual depth. Further studies revealed that ChatGPT is highly proficient in solving logarithmic and exponential equations but faces challenges with derivatives and their applications (Supriyadi and Kuncoro 2023). Similarly, Frieder et al. (2023) found that GPT-4 performs well at the undergraduate level but experiences performance decline at the graduate level, indicating limitations in handling advanced mathematical reasoning.

While AI models have demonstrated the ability to generate human-like responses, their cognitive limitations remain a central debate in AI and education research (Zhai et al. 2024). Despite advancements in pattern recognition and automated reasoning, AI’s ability to generate creative solutions to novel scientific and mathematical problems is still questionable (Dao and Le 2023; Frieder et al. 2023; Rahman and Watanobe 2023; Sandu et al. 2024). Researchers emphasize that achieving educational goals with AI requires not only technological advances but also the integration of well-designed pedagogical approaches and human oversight (Asare et al. 2023; Lake et al. 2016; Parra-Martinez et al. 2023; Rahman and Watanobe 2023; Stanovich 2014).

### 2.3. Higher-Order Thinking

GAI plays a crucial role in supporting higher-order thinking skills, which include analysis, synthesis, and evaluation—key cognitive abilities in mathematical problem-solving (Sternberg 2021). AI-driven systems can identify patterns in large datasets, provide targeted feedback, and offer structured problem-solving strategies to enhance students’ cognitive engagement (Zhai et al. 2020). Personalized AI-driven tutoring models allow students to interact with complex problems at an appropriate difficulty level, promoting conceptual understanding and adaptive learning (Chen et al. 2020; Daher et al. 2023; Kirschner et al. 2006; Ogunleye et al. 2024).

By using deep learning and machine learning algorithms, GAI facilitates pattern recognition, logical inference, and problem decomposition, enabling students to visualize and manipulate abstract mathematical concepts (Bommasani et al. 2021). Additionally, AI-enhanced learning tools help individuals develop metacognitive skills, guiding them through the process of self-regulated learning and critical thinking (Bahroun et al. 2023; Sánchez-Ruiz et al. 2023; Wardat et al. 2023; Yoon et al. 2024). However, while AI can support cognitive growth, it does not inherently foster original thought, raising concerns about its impact on students’ creative problem-solving abilities.

### 2.4. GAI Technologies and Commonsense Problem-Solving

Commonsense problem-solving is the ability to apply logical reasoning and practical judgment to complex, ambiguous situations (Minsky 2006). In AI research, this capacity is essential for developing machine intelligence that can operate effectively in real-world contexts (Zhai et al. 2024). Although GAI systems demonstrate impressive computational capabilities, they often struggle with contextual reasoning and intuitive decision-making (Lake et al. 2016; Levesque 2017). The current AI models lack the nuanced understanding required to interpret unstructured data or make socially informed judgments, limiting their applicability in authentic mathematical reasoning scenarios (Davis and Marcus 2015). Future advancements in AI may address these shortcomings through big data processing and improvements in human-like reasoning frameworks (Weld and Bansal 2019).

### 2.5. Research Gap and Study Contribution

While previous studies have explored AI’s role in tutoring and procedural mathematics learning, limited research has assessed its ability to perform on standardized assessments requiring conceptual reasoning and problem-solving. This study addresses this gap by systematically comparing AI’s performance with human proficiency in NAEP mathematics tasks.

## 3. Materials and Methods

### 3.1. Study Design

This study follows a descriptive and comparative research design, analyzing AI performance in NAEP mathematics assessments and comparing it with student performance based on publicly available aggregate data. The goal is to evaluate the capabilities of GAI models (e.g., ChatGPT-4o and GPT-4) in solving mathematics problems of varying complexity levels, aligning with cognitive load theory. This approach provides insights into AI’s strengths and limitations in conceptual and procedural mathematical reasoning.

### 3.2. Population and Sampling

The population for this study consists of 4th-, 8th-, and 12th-grade students who participated in NAEP assessments. The 4th- and 8th-grade questions were selected from 2022, while the 12th-grade questions were drawn from previous periods (2009–2013) due to limited publicly available data. The NAEP assessments provided a nationally representative sample of students across the U.S., ensuring a robust comparative benchmark (National Center for Education Statistics [NCES] 2023). However, this study does not involve direct human participants; instead, it utilizes aggregate student performance data provided by NAEP to compare against AI-generated responses. Due to privacy restrictions, individual student responses were not accessed. A total of 60 assessment questions were selected based on the following criteria: availability of released NAEP items from The Nation’s Report Card database; coverage of five major mathematical content areas: number properties and operations, measurement, geometry, data analysis and probability, and algebra; and representation of diverse cognitive complexity levels, categorized as low, moderate, and high (National Assessment Governing Board [NAGB] 2022). These selection criteria ensure a balanced and representative sample of mathematics tasks, allowing a systematic comparison of AI-generated responses with NAEP performance trends.

### 3.3. Instruments and Materials

A total of 60 assessment questions (20 questions from each grade level) published by NAEP at the 4th-, 8th-, and 12th-grade levels were used to compare the mathematical problem-solving abilities of ChatGPT-4o and GPT-4 models with those of students. Questions for the 4th and 8th grades were selected from 2022, while the 12th-grade questions were taken from previous periods (2009–2013) due to the unavailability of openly accessible 12th-grade questions from 2022. NAEP regularly evaluates the academic performance of 4th-, 8th-, and 12th-grade students in various areas, including mathematics, reading, U.S. history, science, writing, geography, arts, economics, technology and engineering literacy, foreign languages, and civics (National Center for Education Statistics [NCES] 2023). In recent years, NAEP has increased the use of technology by transitioning to digital assessments and has adopted a broader definition of student performance (National Center for Education Statistics [NCES] 2020). The reason that mathematics was chosen for this study is its critical role in education, as it enhances students’ analytical thinking, problem-solving, and logical inference skills. Mathematics is interconnected with other disciplines at both basic and advanced levels and demands foundational competence in areas such as science, language, engineering, and economics (National Council of Teachers of Mathematics [NCTM] 2014).

The NAEP mathematics assessment consists of five main content areas: number properties and operations, measurement, geometry, data analysis, statistics and probability, and algebra (National Assessment Governing Board [NAGB] 2022). These areas aim to measure the multifaceted nature of mathematics and students’ diverse mathematical skills. NAEP test items are designed to assess both fundamental and complex problem-solving skills. The assessments include questions at low, medium, and high levels of complexity, which help determine students’ cognitive load. These questions assess students’ mathematical performance across a wide range of tasks, from routine operations to abstract thinking and analytical skills. NAEP test items are aligned with cognitive load theory and aim to measure students’ levels of mathematical understanding (Brüggemann et al. 2023; Paas et al. 2010; Prisacari and Danielson 2017). This feature makes NAEP an ideal tool for evaluating our questions, as it assesses a wide range of mathematical skills across varying complexity levels, ensuring the effective use of students’ cognitive capacities in line with cognitive load theory (Sweller 2020). Additionally, NAEP tests provide comprehensive analysis opportunities with reliable, long-term data, offering a robust dataset for examining performance differences across student groups, which supports the validation of our research questions across a broad population.

Of the NAEP test items used in the study, 66.6% (*n* = 40) consisted of 2022 data, while 33.3% (*n* = 20) were drawn from earlier periods (2009–2013). The 2022 NAEP math test differs from previous years in several ways due to the impact of the COVID-19 pandemic. These tests placed more emphasis on mathematical thinking and problem-solving skills. NAEP tests are designed to assess not only students’ ability to recall mathematical facts but also their ability to apply that knowledge in problem-solving. Assessment questions are evaluated along two main dimensions: content area and the level of mathematical complexity. These dimensions reflect both what students know and the complexity of the problems they are capable of solving.

Mathematical complexity is categorized into three levels: low-complexity tasks (which require following basic instructions and usually involve the use of standard math procedures), moderate-complexity tasks (which require flexible thinking and involve solving multi-step problems), and high-complexity tasks (which challenge cognitive abilities and often require abstract reasoning or solving novel problems). The content areas include number properties and operations (computation and understanding number concepts); measurement (the use of tools, application of processes, and understanding of area and volume); geometry (covering spatial reasoning and geometric properties); data analysis; statistics and probability (encompassing graphical displays and statistical concepts); and algebra (representations and relationships) (National Assessment Governing Board [NAGB] 2005). These structures offer an in-depth review of K–12-level mathematics exams and provide a comprehensive assessment of ChatGPT-4o and GPT-4’s mathematical problem-solving competencies. The NAEP exam includes a nationally representative sample of students across various grade levels. In 2022, a total of 116,200 fourth graders from 5780 schools and 111,000 eighth graders from 5190 schools participated in the assessment. In 2013, 92,000 12th graders from 8000 schools took part in the assessment.

Using the NAEP Question Tools (The Nation’s Report Card 2022), researchers collected all available assessment items for each grade level and content classification: 25 selected-response questions, 18 short constructed-response questions, 7 extended constructed-response questions, and 10 multiple-choice questions, making a total of 60 items (see Table 1). The majority of these question formats consist of selected-response items. Selected-response items require students to choose the best answer after reading the question, reflecting on it, or performing calculations. This format is suitable for quickly determining whether students have mastered specific knowledge and skills. Short constructed-response items require students to provide a numerical result, a correct classification, a drawing of a concept, or a brief explanation. Extended constructed-response items require students to address a situation that demands more than just a numerical answer or brief explanation. Multiple-choice items require students to select the correct answer from the given options. These types of questions are used to assess a wide range of topics in a short period of time and are scored based on correct answers.

### 3.4. Data Analysis

#### 3.4.1. Detailed Coding Framework and Procedures for Cognitive Load in NAEP Tasks

**Figure 1:**
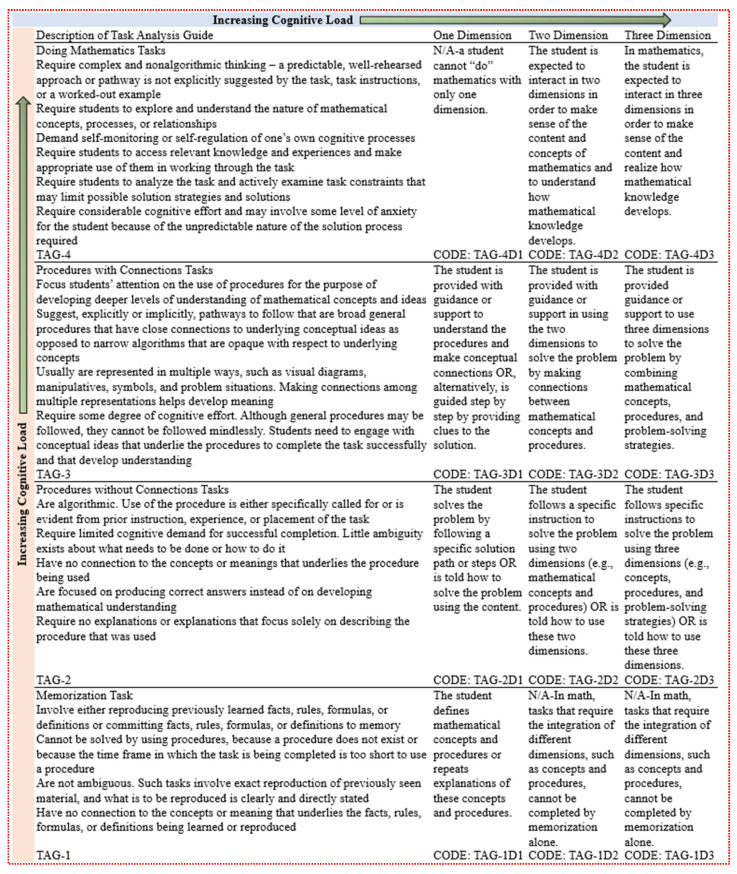
A framework of the cognitive load for the mathematics assessment. Note: Adopted from The Mathematical Task Analysis Guide (Stein et al. 2000), Depth of Knowledge (Webb 2007), and Cognitive Loading in Three-Dimensional NGSS Assessment (Center for Standards, Assessment, and Accountability (CSAA) 2019).

To address the research questions, three mathematics domain experts analyzed and coded the cognitive demands of the NAEP assessment tasks using a framework based on the Mathematical Task Analysis Guide (Stein et al. 2000), Depth of Knowledge (Webb 2007), and Cognitive Loading in Three-Dimensional NGSS Assessment (Center for Standards, Assessment, and Accountability (CSAA) 2019) (see Figure 1).

This framework is used to analyze and classify the cognitive demands of mathematical tasks. Therefore, tasks in mathematics teaching and assessment are designed to encourage higher-level thinking rather than memorization and procedural execution (Center for Standards, Assessment, and Accountability (CSAA) 2019; Stein et al. 2000; Webb 2007). Two dimensions are considered to measure the cognitive demand level of the tasks and to balance basic and higher-order thinking skills. The first dimension progresses from “Memorization” (Task Analysis Guide 1, TAG-1) to “Procedures without Connections Tasks” (Task Analysis Guide 2, TAG-2), “Procedures with Connections Tasks” (Task Analysis Guide 3, TAG-3), and, finally, to “Doing Mathematics” (Task Analysis Guide 4, TAG-4). The second dimension expands the cognitive load from a one-dimensional task to the integration of two dimensions (e.g., TAG-2D2, TAG-3D2, and TAG-4D2) and three dimensions (e.g., TAG-2D3, TAG-3D3, and TAG-4D3). The cognitive load of an item refers to the mental effort and attention students invest while completing a task. The cognitive load fluctuates according to the complexity of the tasks and their problem-solving and information processing requirements (Paas et al. 2010; Sweller 1988; Sweller and Chandler 1991; Webb 2007).

**Figure 2:**
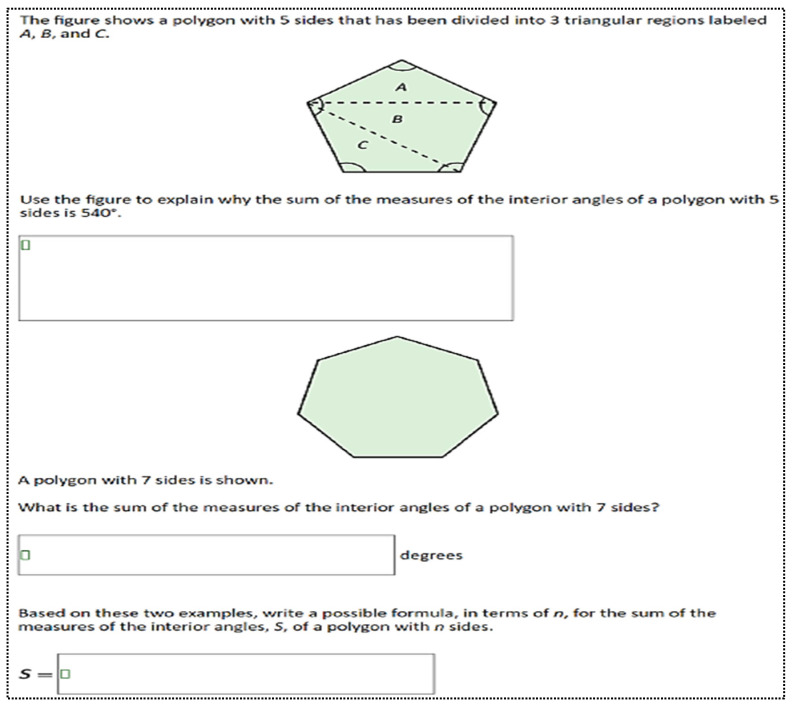
Explain how to produce sounds (NAEP, Mathematic, Grade 8, Year 2022).

The tasks were designed to include various dimensions of cognitive complexity. These tasks are defined by students’ capacity for independent decision-making and their ability to integrate multidimensional information (Zhai and Pellegrino 2023). The cognitive load of a task is measured by the extent to which the student thinks independently and can combine and synthesize different concepts. For example, one of the NAEP math questions used in this study, labeled as 8th grade (2022-8M1 #16 M3873CL), belongs to the geometry category, with a difficulty level indicated as “Hard” (see Figure 2).

This question requires students to demonstrate geometrically that the sum of the interior angles of a pentagon is 540°. The purpose of the question is to help students understand the formula for calculating the sum of the interior angles of polygons and how to apply it to geometric shapes. Students should know that the sum of the interior angles of a triangle is 180°. This information is important in determining the sum of the interior angles of polygons. The pentagon in the question is divided into three triangles (A, B, and C), and since the sum of the interior angles of each triangle is 180°, the total can be calculated as 3 × 180° = 540°. Students can also apply this method more generally to calculate the sum of the interior angles of any n-sided polygon using the formula (*n* − 2) × 180°. Since *n* = 5 for a pentagon, this formula gives (5 − 2) × 180° = 540°.

The goal is for students to make the connection between the sum of the interior angles of triangles and polygons and to calculate the sum by dividing polygons into triangles. In this process, students are expected to establish a relationship between the sum of the interior angles of triangles and the total interior angles of polygons. It is important for students to understand conceptual connections rather than rely on the memorization of formulas. Therefore, the question falls under the category “Procedures with Connections [TAG-3]”. There are three levels in this category (e.g., TAG-3D1, TAG-3D2, and TAG-3D3). In this category, students combine mathematical concepts, procedures, and problem-solving strategies to produce solutions (TAG-3D3). The process of dividing a polygon into triangles and calculating the sum of the interior angles develops logical thinking and problem-solving skills. Therefore, the question is categorized under the “Doing Mathematics [TAG-4]” category. In the third category, students are expected to derive a general formula for the sum of the interior angles of polygons. This process supports students’ skills in generalizing mathematical concepts and deriving formulas. Reaching the formula S = (*n* − 2) × 180° deepens students’ conceptual understanding and enables them to understand how mathematical knowledge develops (TAG-4D3). The three evaluation experts who undertook the coding task were respected professionals with internationally recognized expertise in mathematics education. To ensure consistency in data analysis and interpretation, all assessors were provided with detailed instructions and necessary training. First, several sample questions were selected and coded independently by the raters. Any differences in coding were discussed and resolved until a consensus was reached. This process helped clarify the coding framework and ensured a mutual understanding among the raters. The test items were then assigned to the raters for independent coding. In order to measure the consistency among the raters, the intraclass correlation coefficient (ICC = 0.966, *p* < 0.001) was calculated, indicating a high level of agreement (see Table 2).

#### 3.4.2. Coding Outcomes

Table 3 illustrates how each examined item was coded according to the two cognitive load dimensions. Cognitive load is measured by two primary dimensions: task complexity and dimension level. Task complexity represents the level of difficulty and mental demands of the operations a student performs while solving a problem. The dimension level indicates how much the task requires reflection, problem-solving, or reasoning. Coding these two dimensions helps us understand the cognitive load of each item. To more accurately assess cognitive demands, we multiplied the two dimensions to obtain a single cognitive load score. For example, dimension level 2 requires a moderate level of cognitive processing and involves neither deep nor complex thinking. Dimension level 3 requires higher-order thinking and analytical skills. The student may need to think about abstract concepts, engage in extensive reasoning, or draw conclusions by integrating multiple sources of information. By combining these two levels, we derive a total cognitive load score of 6 (2 × 3 = 6). This score reflects the overall cognitive complexity that the question demands from the student. It also enables us to make quantitative comparisons between items.

#### 3.4.3. Statistical Analysis

To address the research questions, the responses from ChatGPT-4o and GPT-4 were analyzed using a scoring rubric to objectively evaluate response quality. This rubric assessed accuracy based on specific criteria, showcasing individual AI performance and enabling comparisons with human responses. By evaluating AI and human answers to the same questions, the alignment between AI models and human performance was assessed. However, questions requiring visuals or simulations posed challenges for the AI, as these demand cognitive processes beyond the models’ capabilities. Due to their inability to process visual input, such questions were excluded, leaving 60 items for the final comparison.

Another limitation is that access to NAEP student data is restricted due to privacy protections. These protections prevent researchers from accessing sensitive data, such as individual student performance. As a result, researchers were unable to directly compare student responses with AI models. Given these limitations, the available data only include the mean ability scores and the percentages of students who answered each item correctly. These data were calculated using Item Response Theory. This theory provides a model for predicting the difficulty levels of questions and the ability levels of students. The ability levels and the percentage of students who answered each question correctly indicate the difficulty of the questions and how students coped with these challenges. It should be emphasized that this study is a secondary analysis of existing data. The study used data provided by the NCES. The researcher did not directly test the validity of the scores but relied on the existing data.

To address research question (a), we evaluated the problem-solving performances of the ChatGPT-4o and GPT-4 models according to the percentage of students who solved the problems correctly. Our goal is to measure the capabilities of these models by assessing their rankings within the student body. If ChatGPT-4o and GPT-4 successfully solve a problem where “*n*” students have a percentile value of “$s_{n}$”, we assume that these models rank, on average, among the students who successfully answered the same question. To calculate this, we used the formula $s_{n}$/2 + (1 − $s_{n}$) = 1 − $s_{n}$/2. With this approach, we analyzed the problem-solving success of the models by comparing them with student groups. If the models failed to solve a problem, we assumed they ranked at an average level among the students who answered incorrectly, using the formula (1 − $s_{n}$)/2. Thus, we analyzed the ranking of ChatGPT-4o and GPT-4 among the student population based on both correct and incorrect solutions. 

For example, if 76% of students answered a question correctly, we evaluate the performance as 1 − (0.76/2) = 62% when ChatGPT-4o and GPT-4 answered this question correctly. This value indicates that the AI models performed better than 62% of the students. If the AI models answer the question incorrectly, we evaluate their performance as (1 − 0.76)/2 = 12%. This value indicates that the AI models outperformed 12% of the students. This approach allows the models to estimate their ranking within the student population based on both correct and incorrect answers. To address research question (b), the evaluation scores of ChatGPT-4o and GPT-4, the Essential Average Student Performance Score (EASPS), and the cognitive loads of the questions were compared using crosstabulation analysis by grade level. EASPS represents the average ability score of students who correctly answered individual questions. These data are provided by The Nation’s Report Card. Crosstabulation analysis is a method used to examine the relationship between two or more categorical variables (Agresti 2013). This analysis generates a frequency table to examine the distribution between categories and aids in determining whether a relationship exists between two variables (Pallant 2020).

## 4. Results

### 4.1. Can ChatGPT-4o and GPT-4 Surpass Human Performance on NAEP Mathematics Assessments?

**Figure 3:**
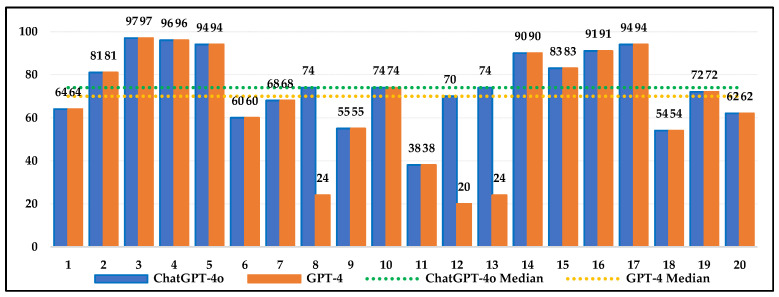
Percentage of students in grade 4 who scored below ChatGPT-4o or GPT-4 for each item.

**Figure 4:**
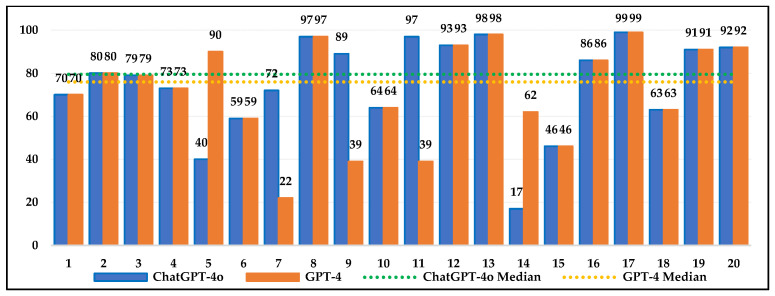
Percentage of students in grade 8 who scored below ChatGPT-4o or GPT-4 for each item.

**Figure 5:**
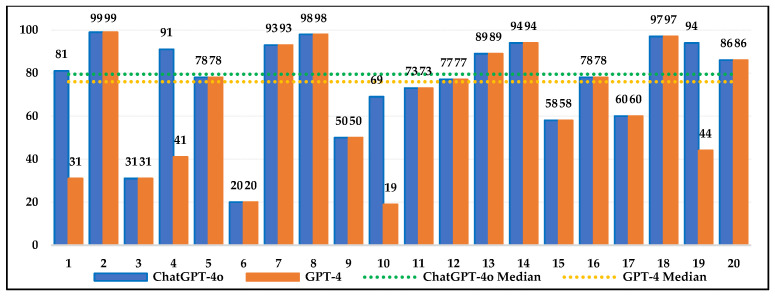
Percentage of students in grade 12 who scored below ChatGPT-4o or GPT-4 for each item.

Figure 3, Figure 4 and Figure 5 show the percentages of 4th-, 8th-, and 12th-grade students who scored below ChatGPT-4o and GPT-4 on each question. ChatGPT-4o and GPT-4’s requests for additional information in some items resulted in them being evaluated as missing data. According to the median values, ChatGPT-4o outperformed 74% of 4th-grade students, 80% of 8th-grade students, and 80% of 12th-grade students. For GPT-4, the values were 70%, 76%, and 75%, respectively. These findings suggest that both AI models outperformed the majority of students. The median values indicate that ChatGPT-4o outperforms GPT-4, suggesting it can provide results comparable to or better than student responses, making it a potentially effective tool in education. The issue of missing data caused by requests for additional information highlights the weaknesses and limitations of AI models more clearly. This is an important factor to consider for more effective AI use in education. Besides the median values, the interquartile range (IQR) values, which show the spread of performance, were examined. The IQR values for ChatGPT-4o are 27%, 29%, and 27% for the 4th, 8th, and 12th grades, respectively, while, for GPT-4, they are 36%, 30%, and 47%. ChatGPT-4o’s lower IQR values (27% for the 4th and 12th grades and 29% for the 8th grade) indicate that the model performs more consistently and provides more predictable results in relation to student performance. GPT-4’s higher IQR values for the 4th and 12th grades (36% and 47%) indicate greater variability in performance, with the potential for both very high and very low results on some questions. Large fluctuations in the performance of GPT-4 were observed, especially in grade 12. For the 8th grade, the IQR values of both models are similar (29% and 30%), indicating a comparable spread in their performance.

### 4.2. How Well Do ChatGPT-4o and GPT-4 Address Cognitive Demands on NAEP Mathematics Assessments Compared to Humans?

The analysis for the research question relied on Kendall’s τb correlation results to examine how ChatGPT-4o and GPT-4 respond to cognitive demand levels in comparison to average students on the NAEP mathematics exams. This analysis was conducted at the 4th-, 8th-, and 12th-grade levels, and the interactions among the three components (e.g., students, ChatGPT-4o, and GPT-4) with the cognitive load were examined (see Table 4). In Grade 4, there was a positive correlation between EASPS and cognitive load ($\tau_{b}$(4) = 0.664, *p* < 0.001, 95% CI [0.661, 0.668]), indicating that, as the cognitive demand increases, students require a higher level of ability to answer questions correctly. This finding suggests that more challenging questions require enhanced information processing and problem-solving skills. On the other hand, ChatGPT-4o ($\tau_{b}$(4) = −0.502, *p* < 0.01, 95% CI [−0.507, −0.498]) and GPT-4 ($\tau_{b}$(4) = −0.469, *p* < 0.01, 95% CI [−0.474, −0.464]) show a significant negative correlation with the cognitive load, indicating a decrease in model performance as the cognitive demand increases. The findings reveal that AI models struggle with complex and analytical questions, and their accuracy decreases as the difficulty level rises.

In Grade 8, a significant correlation between EASPS and cognitive load was observed ($\tau_{b}$(8) = 0.557, *p* < 0.01, 95% CI [0.152, 0.802]). This finding suggests that 8th-grade students’ performance decreases as the cognitive load increases, indicating greater sensitivity to cognitive demands. As the cognitive load increases, students experience more difficulty, leading to a decrease in performance. On the other hand, ChatGPT-4o ($\tau_{b}$(8) = −0.412, *p* < 0.05, 95% CI [−0.722, 0.037]) and GPT-4 ($\tau_{b}$(8) = −0.430, *p* < 0.05, 95% CI [−0.733, 0.015]) exhibited a significant negative correlation with cognitive load. These results indicate that AI models show stable performance despite increasing the cognitive load, with the performance remaining stable or slightly improving as the cognitive load increases. In Grade 12, a significant correlation between EASPS and cognitive load was observed ($\tau_{b}$(12) = 0.469, *p* < 0.01, 95% CI [0.034, 0.755]). This finding shows that, as the cognitive load increases, students need to exert more effort in complex tasks, and this effort is crucial for success. This suggests that, as the cognitive load increases, students undergo adaptation processes to enhance their performance. On the other hand, ChatGPT-4o ($\tau_{b}$(12) = −0.332, *p* > 0.05, 95% CI [−0.675, 0.128]) and GPT-4 ($\tau_{b}$(12) = −0.280, *p* > 0.05, 95% CI [−0.643, 0.184]) did not exhibit a statistically significant correlation with cognitive load. These findings suggest that the correlations obtained for the AI models are not generalizable and have limited reliability.

In summary, students require greater information processing and problem-solving skills to solve more complex tasks as the cognitive load increases. While the increased cognitive load in the 4th and 8th grades leads to a decrease in students’ performance, 12th-grade students adapt by putting in more effort. In contrast, AI models (ChatGPT-4o/GPT-4) appear unaffected by increases in the cognitive load, and their performance remains largely constant, suggesting that AI’s capacity to handle complex cognitive demands differs from that of humans.

## 5. Discussion

This study showed that the AI models ChatGPT-4o and GPT-4 outperformed the majority of students who answered each question on the NAEP math assessments. It was also observed that both models demonstrated notable consistency in their performance. Both models exhibited notable consistency in their performance, with agreement rates of 85% for the 4th grade, 75% for the 8th grade, and 80% for the 12th grade. This consistency suggests that the AI models processed and answered mathematics problems in a systematic manner, showing high levels of accuracy and comprehension across different grade levels.

### 5.1. Cognitive Load, Grade Levels, and AI Performance

Research findings suggest that, as cognitive demands increase on NAEP mathematics assessments, student performance tends to decline, with younger students being more sensitive to cognitive load variations. Fourth-grade students exhibited the strongest sensitivity to increased problem complexity, demonstrating a greater need for structured problem-solving strategies. Although the 8th and 12th graders also experienced the effects of cognitive load, their performance showed more stability at higher levels of complexity.

However, ChatGPT-4o and GPT-4 did not exhibit the same level of sensitivity to cognitive demands, particularly at the 12th-grade level, where performance remained stable regardless of task complexity. While AI performance was moderately affected by cognitive load at the 4th- and 8th-grade levels, its overall stability suggests that AI models do not experience the same cognitive constraints as human learners. One notable finding is that, as the grade level increases, AI models’ performance appears to be less influenced by task complexity. This suggests that AI may handle abstract reasoning more effectively at advanced levels, where structured computational thinking is required.

These findings provide important insights into the role of AI models in educational settings. At the elementary level, where students rely more on structured cognitive scaffolding, AI may serve as a guided problem-solving tool. At higher grade levels, AI models might be better suited to handling abstract and complex problems independently. However, the study also highlights limitations in AI’s adaptability to human-like reasoning processes, reinforcing the need for a balanced integration of AI into educational environments.

### 5.2. AI as a Supplementary Educational Tool

AI models ChatGPT-4o and GPT-4 outperform most students on NAEP math assessments, reinforcing their potential as supplementary tools in mathematics education. However, it is crucial to recognize their limitations in complex problem solving, creative thinking, and critical analysis. While these models are highly efficient at procedural tasks and pattern recognition, they struggle with generalization and novel problem-solving scenarios (Davis and Marcus 2015; Floridi and Chiriatti 2020; Levesque 2017; Zhai et al. 2024). Human reasoning remains more flexible and adaptable, particularly in open-ended problem contexts where judgment and conceptual understanding are essential.

Additionally, the study highlights the importance of ethical and responsible AI use in education. While AI has the potential to accelerate access to knowledge, personalize learning, and enhance problem-solving efficiency, its risks must be addressed (Pesovski et al. 2024; Wardat et al. 2023; Zhai et al. 2020). These risks include overreliance on AI tools, biases in AI-generated responses, and the potential for students to disengage from active learning. Therefore, educators must establish guidelines for AI integration, ensuring that students actively engage in critical thinking rather than passively relying on AI-generated solutions.

### 5.3. Balancing AI and Human Guidance in Mathematics Education

While AI models excel at solving well-structured problems, their role as an educational tool must be carefully balanced with teacher guidance and student engagement strategies. The relationship between cognitive load and AI performance suggests that AI models may be particularly effective at lower grade levels for structured tasks, whereas higher grade levels may require more human-driven instructional strategies (Wardat et al. 2023).

AI-supported teaching tools can successfully reinforce foundational mathematical concepts, but higher-order thinking and complex problem-solving skills still require human intervention (Chen et al. 2020). While cognitive load can be beneficial for deep learning, excessive complexity may hinder student success, emphasizing the importance of well-calibrated instructional design. Teachers should carefully balance the cognitive difficulty of AI-assisted tasks to ensure that students are adequately challenged without being overwhelmed (Asare et al. 2023; Rahman and Watanobe 2023). Achieving this balance will optimize student learning and foster deeper conceptual understanding.

### 5.4. Rethinking Assessment Strategies in an AI-Driven Era

Findings from this study indicate that current assessment methods must evolve to reflect the changing nature of learning in AI-supported classrooms. Standardized assessments such as NAEP have traditionally been designed to measure human problem-solving abilities under conventional testing conditions. However, AI’s ability to solve these problems efficiently challenges the effectiveness of such assessments in evaluating conceptual understanding and reasoning skills (Zhai and Wiebe 2023).

Given these findings, innovative assessment strategies should be developed to emphasize creativity, conceptual reasoning, and applied knowledge. To maximize AI’s impact on student learning, its limitations in high-cognitive load problem-solving must be addressed by integrating human-led instructional techniques. AI should not replace human learning processes but rather enhance and support them, ensuring that students retain critical and independent thinking skills (Chen et al. 2020; Paas and van Merriënboer 2020).

### 5.5. Limitations and Trends for Future Research

While this study provides valuable insights into AI’s performance in mathematics education, it also presents certain limitations. One key limitation is the restricted availability of direct student response data from NAEP, which limited the study to aggregate performance comparisons. Future research can explore alternative datasets, such as TIMSS and PISA, to enable a more detailed analysis of AI performance relative to student cognitive strategies. Another consideration is AI’s familiarity with standardized test question structures, which may have influenced performance outcomes. Since AI models are trained on extensive datasets, they may have been exposed to question formats similar to those used in NAEP assessments. Future studies should explore AI performance on entirely novel problem sets, assessing its ability to generalize mathematical reasoning beyond pattern recognition.

## 6. Conclusions

The results of this study indicate that ChatGPT-4o and GPT-4 are capable of solving standardized mathematics problems at a level that surpasses most student performance. However, AI’s ability to generalize mathematical reasoning and solve high-cognitive load problems remain limited. These findings emphasize the need for strategic AI integration in education, ensuring that AI functions as a complementary tool rather than a replacement for traditional learning methods.

As AI continues to advance, its impact on curriculum design, instructional methodologies, and student learning outcomes will require ongoing investigation. Future research should explore how AI can support higher-order thinking, promote engagement, and enhance conceptual learning. By aligning AI with effective pedagogical strategies, educators can leverage AI’s potential while preserving essential cognitive skills such as creativity, critical thinking, and independent problem-solving.

## Figures and Tables

**Table 1:** Format and number of items in the NAEP mathematics assessment.

	Grade Level	SR ^1^	SCR ^2^	ECR ^3^	MC ^4^	Sum (SR + SCR + ECR + MC)
Number properties and operations	4	3	1	1	-	5
	8	3	2	-	-	5
	12	-	-	1	1	2
Total		6	3	2	1	12
Measurement	4	5	1	-	-	6
	8	2	2	-	-	4
	12	-	-	-	2	2
Total		7	3	0	2	12
Geometry	4	2	1	-	-	3
	8	2	1	1	-	4
	12	-	1	2	2	5
Total		4	3	3	2	12
Data analysis, statistics, and probability	4	2	-	1	-	3
	8	2	2	-	-	4
	12	-	3	-	2	5
Total		4	5	1	2	12
Algebra	4	2	1	-	-	3
	8	2	1	-	-	3
	12	-	2	1	3	6
Total		4	4	1	3	12
Grand total	Sum	25	18	7	10	60

^1^ SR: Selected Response; ^2^ SCR: Short Constructed Response, ^3^ ECR: Extended Constructed Response, and ^4^ MC: Multiple Choice.

**Table 2:** Intraclass correlation coefficient of the cognitive load rating of the items.

	Intraclass Correlation ^b^	95% Confidence Interval		F Test with True Value 0		
		Lower Bound	Upper Bound	Value	${df}_{1}$	${df}_{2}$
Single rater measures	0.905 ^a^	0.888	0.917	29.827	59	120
Average measure for all raters	0.966 ^c^	0.948	0.978	27.827	59	120

^a^ Single rater measure. ^b^ Two-way mixed model, absolute agreement. ^c^ Measure based on the average of raters.

**Table 3:** Cognitive load of the NAEP assessment tasks.

ID	Grade	Subject	Difficulty	Type	Cognitive Load		
					Dimension	Task	Aggregated
1	4	Number Properties	Easy	SCR	1	2	2
2	4	Number Properties	Medium	SR	3	2	6
3	4	Number Properties	Medium	SR	3	3	9
4	4	Number Properties	Hard	SR	3	3	9
5	4	Number Properties	Hard	ECR	3	2	6
6	8	Number Properties	Easy	SR	1	2	2
7	8	Number Properties	Medium	SCR	2	2	4
8	8	Number Properties	Medium	SR	2	2	4
9	8	Number Properties	Medium	SR	3	2	6
10	8	Number Properties	Hard	SCR	3	3	9
11	12	Number Properties	Hard	MC	2	2	4
12	12	Number Properties	Hard	ECR	3	3	9
13	4	Measurement	Easy	SR	2	2	4
14	4	Measurement	Easy	SR	2	2	4
15	4	Measurement	Easy	SR	3	2	6
16	4	Measurement	Medium	SR	2	2	4
17	4	Measurement	Medium	SR	3	2	6
18	4	Measurement	Hard	SCR	3	3	9
19	8	Measurement	Easy	SCR	2	2	4
20	8	Measurement	Medium	SR	2	3	6
21	8	Measurement	Hard	SR	3	3	9
22	8	Measurement	Hard	SCR	2	2	4
23	12	Measurement	Hard	MC	3	3	9
24	12	Measurement	Hard	MC	3	3	9
25	4	Geometry	Medium	SR	2	2	4
26	4	Geometry	Medium	SCR	2	2	4
27	4	Geometry	Hard	SR	3	3	9
28	8	Geometry	Easy	SR	2	3	6
29	8	Geometry	Hard	SR	3	3	9
30	8	Geometry	Hard	SCR	3	3	9
31	8	Geometry	Hard	ECR	3	3	9
32	12	Geometry	Medium	MC	2	3	6
33	12	Geometry	Medium	MC	3	3	9
34	12	Geometry	Hard	SCR	3	2	6
35	12	Geometry	Hard	ECR	3	4	12
36	12	Geometry	Hard	ECR	3	3	9
37	4	Data Analysis	Hard	SR	2	2	4
38	4	Data Analysis	Hard	SR	3	2	6
39	4	Data Analysis	Hard	ECR	3	3	9
40	8	Data Analysis	Easy	SR	2	2	4
41	8	Data Analysis	Medium	SCR	2	3	6
42	8	Data Analysis	Hard	SR	3	2	6
43	8	Data Analysis	Hard	SCR	3	3	9
44	12	Data Analysis	Easy	MC	3	3	9
45	12	Data Analysis	Medium	MC	2	3	6
46	12	Data Analysis	Medium	SCR	3	3	9
47	12	Data Analysis	Hard	SCR	3	3	9
48	12	Data Analysis	Hard	SCR	3	4	12
49	4	Algebra	Easy	SR	1	2	2
50	4	Algebra	Medium	SR	2	3	6
51	4	Algebra	Medium	SCR	2	3	6
52	8	Algebra	Easy	SR	3	2	6
53	8	Algebra	Medium	SR	3	3	9
54	8	Algebra	Hard	SCR	3	4	12
55	12	Algebra	Easy	MC	2	2	4
56	12	Algebra	Medium	SCR	3	3	9
57	12	Algebra	Medium	MC	3	2	6
58	12	Algebra	Hard	SCR	3	4	12
59	12	Algebra	Hard	ECR	3	3	9
60	12	Algebra	Hard	MC	3	3	9

**Table 4:** Problem-solving abilities of the students, ChatGPT-4o, and GPT-4 by cognitive demand.

				95% Confidence Intervals (2-Tailed) ^a^	
Grade Level	Variables_CL	$\mathrm{Kendall}’s\;\tau_{b}$	Significance (2-Tailed)	Lower	Upper
4	EASPS_CL	0.664 ***	0.000	0.661	0.668
	ChatGPT-4o-score_CL	−0.502 **	0.005	−0.507	−0.498
	GPTscore_CL	−0.469 **	0.008	−0.474	−0.464
8	EASPS_CL	0.557 **	0.001	0.152	0.802
	ChatGPT-4o-score_CL	−0.412 *	0.021	−0.722	0.037
	GPTscore_CL	−0.430 *	0.015	−0.733	0.015
12	EASPS_CL	0.469 **	0.009	0.034	0.755
	ChatGPT-4o-score_CL	−0.332	0.066	−0.675	0.128
	GPTscore_CL	−0.280	0.121	−0.643	0.184

* *p* < 0.05, ** *p* < 0.01, and *** *p* < 0.001, ^a^ Estimation is based on Fisher’s r-to-z transformation. EASPS: Essential Average Student Performance Score; CL: cognitive load.

## Data Availability

The data supporting the findings of this study are publicly available. NAEP mathematics assessment items and scoring criteria were accessed from publicly accessible resources provided by the National Center for Education Statistics (NCES). The generative AI outputs produced by ChatGPT-4o and GPT-4 based on these assessments are also available upon reasonable request. For further details on accessing NAEP data, please visit https://www.nationsreportcard.gov/nqt/searchquestions.

## Abbreviations

The following abbreviations are used in this manuscript:

AI	Artificial Intelligence
EASPS	Essential Average Student Performance Score
ECL	Extraneous Cognitive Load
GAI	Generative Artificial Intelligence
GPT	Generative Pre-trained Transformer
ICC	Intraclass Correlation Coefficient
ICL	Intrinsic Cognitive Load
IQR	Interquartile Range
IRT	Item Response Theory
MC	Multiple Choice
NAEP	National Assessment of Educational Progress
NAGB	National Assessment Governing Board
NCES	National Center for Education Statistics
NCTM	National Council of Teachers of Mathematics
NGSS	Next Generation Science Standards
SCR	Short Constructed Response
SR	Selected Response
TAG	Task Analysis Guide
